# State-level nutritional disparities and policy priorities in India

**DOI:** 10.3389/fnut.2026.1902541

**Published:** 2026-09-08

**Authors:** Mayadhar Sethy, Sandhya R. Mahapatro

**Affiliations:** Nabakrushna Choudhury Center for Development Studies, Bhubaneswar, Odisha, India

**Keywords:** child undernutrition, spatial analysis, NFHS-6, India, nutritional inequality, dietary vulnerability

## Abstract

**Background:**

Nutritional inequality persists in India despite sustained economic growth, expanding healthcare infrastructure, and large-scale social welfare programs. National averages mask substantial subnational disparities, concealing deep inequalities that continue to shape nutritional outcomes across States and Union Territories. This study quantifies nutritional inequality across Indian States and Union Territories using the latest NFHS-6 factsheet (2023–24) data, integrating multidimensional and spatial methods to provide a comprehensive evidence base for geographically targeted nutrition policy.

**Methods:**

Data from 36 States/UTs (excluding Manipur) were analyzed using an ecological, cross-sectional, and comparative research design. Outcome indicators included child stunting, wasting, and underweight prevalence, which were standardized and aggregated into a Child Undernutrition Burden Index (CUBI). A Dietary Vulnerability Index (DVI) was also constructed using standardized indicators of dietary diversity, nutrient-rich food consumption, and household food insecurity. Inequality was assessed via coefficient of variation, Theil decomposition, and Moran's *I* spatial autocorrelation. Advanced methods included quantile regression to examine heterogeneous associations across burden percentiles and spatial Durbin models to assess direct, indirect, and total spillover effects across neighboring states.

**Results:**

National prevalence was 29.3% for stunting, 19.0% for wasting, and 31.8% for underweight. Jharkhand (CUBI = 1.35), Gujarat (0.94), and Bihar (0.81) had the highest burden, while Goa (−1.45), Kerala (−1.32), and Tamil Nadu (−1.21) had the lowest. Significant spatial autocorrelation (Moran's *I* = 0.47, *p* < 0.01) indicated clustering in eastern and central India. Female education showed stronger protective associations in high-burden states (β=-0.487) than in low-burden states (β=-0.346). The spatial Durbin model identified significant spillovers (ρ = 0.413, *p* < 0.01), including direct (β=-0.362) and indirect (β=-0.184) effects of female education. Within-region inequality accounted for 61.4% of total inequality. Female education, sanitation, and women's empowerment showed the strongest protective associations, explaining 64% of state-level CUBI variation. DVI was strongly associated with CUBI (ρ = 0.842, *p* < 0.001). All findings represent ecological associations and should not be interpreted as individual-level causal effects.

**Conclusion:**

India's nutritional transition remains characterized by persistent spatial clustering and substantial within-region inequality. State-level priorities should focus on female education, sanitation, dietary diversity, and women's empowerment, while CUBI, quantile regression, and spatial spillover evidence can guide targeted interventions and coordinated regional nutrition policies.

## 1 Introduction

### 1.1 Background and context

Nutrition is a fundamental determinant of human development, influencing physical growth, cognitive performance, educational attainment, labor productivity, and long-term health outcomes (1, 2). Adequate nutrition contributes substantially to human capital formation and socioeconomic development, whereas poor nutritional status undermines individual wellbeing and broader national progress (3–5). Despite sustained economic growth, expanding healthcare infrastructure, and large-scale welfare programmes, India continues to face substantial nutritional challenges, including child undernutrition, micronutrient deficiencies, and an increasing burden of overweight and obesity (6–8).

Over the past two decades, major initiatives such as Integrated Child Development Services (ICDS), the National Health Mission (NHM), POSHAN Abhiyaan, and Anemia Mukt Bharat have contributed to improvements in maternal and child health, institutional deliveries, immunization, and selected nutrition indicators (9, 10). However, progress remains uneven across States and Union Territories (UTs), reflecting differences in socioeconomic development, education, healthcare access, dietary practices, sanitation, and governance (11, 12). Consequently, national averages may conceal substantial subnational inequalities that continue to influence nutritional outcomes.

Understanding these disparities requires moving beyond aggregate national estimates toward geographically disaggregated analysis. States with broadly comparable economic conditions may nevertheless experience markedly different nutritional outcomes because of variations in maternal education, sanitation, dietary diversity, healthcare access, and social protection (13). A subnational perspective can identify areas experiencing disproportionate nutritional burdens and support geographically targeted interventions tailored to local determinants (14, 15). Spatial analysis is therefore increasingly important for understanding the distribution and clustering of malnutrition.

The National Family Health Survey (NFHS) is India's most comprehensive source of demographic, health, and nutrition information (6, 16). Since its inception in 1992–93, successive NFHS rounds have generated nationally representative evidence on fertility, mortality, maternal and child health, nutrition, and socioeconomic conditions. The recently released NFHS-6 (2023–24) provides an important opportunity to reassess contemporary nutritional inequalities across India. Standardized State and UT fact sheets facilitate systematic comparison of nutritional indicators and examination of regional disparities (17).

NFHS-6 is particularly relevant amid India's continuing nutrition transition, in which persistent undernutrition coexists with rising overweight, obesity, and diet-related non-communicable diseases (8, 18). Among children, stunting reflects chronic nutritional deprivation, wasting indicates acute nutritional stress, and underweight captures elements of both conditions (19, 20). In NFHS-6, however, anemia indicators were not consistently available across State and UT fact sheets, limiting their incorporation into the composite measure. Accordingly, the Child Undernutrition Burden Index (CUBI) developed in this study focuses specifically on child anthropometric failure rather than the full spectrum of nutritional deprivation.

Contemporary nutrition research recognizes that nutritional outcomes emerge from interacting individual, household, community, and institutional factors (3, 21). Maternal education, sanitation, healthcare access, gender relations, environmental conditions, and economic opportunities are important correlates of nutritional status (13, 22). Malnutrition should therefore be understood not simply as inadequate food consumption but as an expression of broader social, economic, environmental, and developmental inequalities (23, 24).

### 1.2 Literature review and research gaps

Research on nutritional inequality in India has expanded substantially through successive NFHS rounds. NFHS-3 documented pronounced interstate disparities in child undernutrition, while NFHS-4 and NFHS-5 showed gradual national improvements alongside persistent regional inequalities (6, 16, 23, 25). International evidence from South Asia and sub-Saharan Africa similarly emphasizes spatial analysis for understanding geographically concentrated malnutrition (15, 26–28). Methodological advances in spatial epidemiology and multidimensional indices have further strengthened the assessment of nutritional deprivation (29, 30).

Several studies have advanced understanding of India's nutritional inequalities. Kim et al. (14) developed multidimensional nutrition burden indices using NFHS-4 state-level data, while Patil et al. (31) identified persistent undernutrition hotspots in central and eastern India using NFHS-4 and NFHS-5 fact sheets. Dandona et al. (17) demonstrated substantial interstate heterogeneity in India's epidemiological transition, while Sethy and Mahapatro (32) contributed evidence on dietary diversity, structural inequalities, and spatial relationships.

Recent NFHS-5-based research further confirms significant geographical clustering of stunting, wasting, and underweight and highlights maternal, socioeconomic, and spatial determinants (33). Regional disparities in minimum dietary diversity among children aged 6–23 months have also been documented, with greater inadequacy in central and northern districts than in several southern and northeastern areas (34). Spatial regression evidence similarly demonstrates substantial regional variation in dietary patterns and nutrition-related outcomes (35). These findings provide important contextual support for interpreting CUBI and DVI as indicators of geographically differentiated nutritional vulnerability.

The observed state-level heterogeneity is also consistent with broader evidence of spatial variation in India's social, environmental, and economic development. Studies of SDG performance demonstrate considerable differences across States and Union Territories, particularly regarding water, sanitation, urban sustainability, and broader development outcomes (36–38).

Despite these advances, the present study addresses several important gaps: (1) It is among the first to utilize NFHS-6 (2023–24) data; (2) It integrates multiple advanced analytical methods within a unified framework; (3) It explicitly distinguishes between child undernutrition (CUBI) and dietary vulnerability (DVI); (4) Quantile regression reveals how ecological associations vary across the burden distribution; (5) Spatial Durbin Model results provide evidence of spatial spillover effects; and (6) Robustness checks confirm stability across alternative specifications.

Among the correlates of nutritional inequality, maternal education consistently emerges as one of the strongest ecological correlates of child nutritional outcomes. State-level studies have shown that jurisdictions with higher female educational attainment tend to have lower prevalence of child undernutrition. Educated mothers are more likely to adopt appropriate feeding practices, utilize healthcare services, and maintain hygienic environments, thereby improving child nutrition (24, 39). The education-nutrition relationship can be represented as:


$$\begin{array}{l} \frac{\partial S}{\partial E}=\beta_{E}+\beta_{EH}H+\beta_{EF}F+\varepsilon \end{array}$$ (1)

where S denotes nutritional status, E represents maternal education, H captures health knowledge, F reflects feeding practices, and ε denotes unobserved factors. Empirical studies suggest that each additional year of maternal education is significantly associated with reduced likelihood of stunting and underweight among children (13, 22). However, these individual-level relationships should not be directly inferred from state-level ecological associations; the present study examines macro-level structural associations rather than individual behavioral mechanisms.

Sanitation has also received considerable attention as a correlate of nutritional outcomes. Research has demonstrated strong associations between open defecation and child stunting in India (11, 12). Poor sanitation contributes to environmental enteropathy, a condition characterized by chronic intestinal inflammation and impaired nutrient absorption (12). This relationship can be expressed as:


$$\begin{array}{l} S_{i}=\gamma_{0}+\gamma_{1}San_{i}+\gamma_{2}X_{i}+\mu_{i} \end{array}$$ (2)

where San_*i*_ denotes sanitation coverage, X_*i*_ represents other explanatory variables, and μ_*i*_ is the error term. Empirical estimates indicate that improvements in sanitation are associated with substantial reductions in stunting prevalence in high-burden settings (11).

Dietary diversity constitutes another critical correlate of nutritional wellbeing. The Minimum Dietary Diversity for Women (MDD-W) indicator developed by FAO measures the consumption of diverse food groups and serves as a proxy for micronutrient adequacy (40). Numerous studies in India have documented positive associations between dietary diversity and nutritional outcomes (32, 41). The relationship is frequently modeled using logistic regression:


$$\begin{array}{l} \ln\left(\frac{p_{i}}{1-p_{i}}\right)=\alpha +\beta_{1}D_{i}+\beta_{2}X_{i}+\epsilon_{i} \end{array}$$ (3)

where p_*i*_ denotes the probability of nutritional deficiency, D_*i*_ represents dietary diversity, and X_*i*_ is a vector of control variables. Evidence suggests that the benefits of dietary diversity increase rapidly up to a threshold level before exhibiting diminishing marginal returns (42).

Spatial analyses have further revealed significant geographical clustering of nutritional outcomes. Studies employing Bayesian geostatistical models, spatial scan statistics, and multilevel frameworks consistently identify hotspots of undernutrition in central and eastern India (24, 27, 28). The decomposition of nutritional inequality across geographic scales can be expressed as:


$$\begin{array}{l} Var\left(S\right)=\sigma_{state}^{2}+\sigma_{district}^{2}+\sigma_{community}^{2}+\sigma_{household}^{2}+\sigma_{e}^{2} \end{array}$$ (4)

where each variance component represents the contribution of a specific spatial level to overall inequality (43). Existing evidence suggests that state- and district-level factors account for a substantial proportion of variation in nutritional outcomes (31).

Researchers have increasingly adopted composite measures to capture multidimensional nutritional deprivation. One prominent example is the Composite Index of Anthropometric Failure (CIAF), which integrates multiple forms of child undernutrition into a single metric (14). The CIAF framework can be expressed as:


$$\begin{array}{l} CIAF_{i}=\overset{6}{\underset{j\;=\;1}{\sum }}w_{j}I_{ij} \end{array}$$ (5)

where I_*j*_ indicates membership in different anthropometric failure categories and w_*j*_ represents corresponding weights. More recent studies have employed principal component analysis and factor analysis to derive multidimensional nutrition indices that better capture overlapping forms of deprivation (29, 30). The present study builds on this tradition by constructing a Child Undernutrition Burden Index (CUBI) specifically focused on child anthropometric indicators, explicitly distinguishing it from the broader concept of nutritional deprivation that would include adult undernutrition, anemia, and dietary indicators.

Despite substantial advances, several gaps remain. Most existing studies rely on NFHS-4 or NFHS-5 data, leaving limited evidence based on NFHS-6. Advanced methodologies such as spatial Durbin models, quantile regression, and multidimensional decomposition remain underutilized. Additionally, the heterogeneous ecological associations across the nutritional burden distribution have not been adequately examined.

**Study strengths:** this study offers several notable strengths. (1) It is among the first to analyze nutritional inequality using NFHS-6 data. (2) It integrates multiple advanced analytical methods within a unified framework. (3) The construction of the Child Undernutrition Burden Index (CUBI) and DVI allows for multidimensional ranking, with CUBI specifically capturing child anthropometric failure. (4) Quantile regression reveals how ecological associations vary across the burden distribution. (5) Spatial Durbin Model results provide evidence of spatial spillover effects.

### 1.3 Research questions and hypotheses

Based on the identified research gaps, this study addresses the following research questions:

- RQ1: How is nutritional deprivation distributed across Indian States and Union Territories, and does it exhibit significant spatial clustering?
- RQ2: What is the relative contribution of within-region vs. between-region variation to overall nutritional inequality?
- RQ3: What structural factors are most strongly ecologically associated with nutritional burden, and do these associations vary across the burden distribution?
- RQ4: Are there significant spatial spillover effects in nutritional outcomes across neighboring states?

The following testable hypotheses guide the empirical analysis:

- H1: Nutritional deprivation exhibits significant positive spatial autocorrelation, with states in eastern and central India displaying higher CUBI scores.
- H2: Within-region inequality contributes more to overall nutritional inequality than between-region variation.
- H3: Female education, sanitation coverage, and women's empowerment show strong negative ecological associations with CUBI at the state level.
- H4: The ecological associations of structural determinants with nutritional burden vary across the burden distribution, with stronger effects observed in high-burden states.

### 1.4 Research objectives

The present study adopts a multidimensional perspective and integrates multiple indicators of nutritional deprivation, including child stunting, wasting, and underweight prevalence. Rather than evaluating these indicators independently, the study examines their combined influence on nutritional vulnerability across India. Furthermore, evidence from spatial epidemiology suggests that nutritional outcomes frequently exhibit geographical clustering because neighboring regions often share environmental characteristics, food systems, socioeconomic structures, and institutional arrangements. Identifying such spatial patterns can provide valuable insights into the structural ecological correlates of malnutrition and support more effective resource allocation.

Accordingly, this study seeks to: (1) quantify nutritional inequalities across States and UTs using NFHS-6 indicators; (2) develop a multidimensional framework incorporating composite indices, spatial analysis, ranking methods, and advanced econometric techniques; (3) examine heterogeneous ecological associations using quantile regression; (4) assess spatial spillover effects using spatial Durbin models; and (5) translate findings into actionable policy recommendations. By moving beyond descriptive national averages and examining nutritional disparities through a state-level lens, the study contributes to a more nuanced understanding of nutritional inequality and provides evidence to support geographically targeted nutrition policies in India.

## 2 Materials and methods

### 2.1 Study area

India consists of 28 States and 8 Union Territories (UTs), characterized by substantial heterogeneity in demographic composition, socioeconomic development, dietary patterns, environmental conditions, and health outcomes. Such diversity makes India an appropriate setting for examining spatial and regional nutritional inequalities. Moreover, because States and UTs are the primary administrative units responsible for implementing nutrition and health programs, state-level analysis is highly relevant for policy formulation and resource allocation.

### 2.2 Data source

This study utilizes publicly available data from the National Family Health Survey-6 (NFHS-6), 2023–24, conducted by the International Institute for Population Sciences (IIPS) with national and international partners. NFHS is India's largest nationally representative household survey, providing comprehensive information on fertility, mortality, maternal and child health, nutrition, and healthcare utilization. It employs a multistage stratified sampling design and maintains high response rates, supporting reliable and representative estimates (6, 16, 25).

The analysis uses officially released NFHS-6 fact sheets for India and individual States/UTs. All 36 available States/UTs were included, except Manipur because complete data were unavailable at the time of analysis. As district-level NFHS-6 fact sheets had not yet been released, the analysis is restricted to state-level aggregates; future research should extend the analysis to districts when data become available (31).

**Justification for using NFHS fact sheets:** previous studies have successfully used NFHS fact sheets for comparative state-level analysis, including multidimensional nutrition indices and spatial clustering (14, 31), as well as epidemiological transitions (17). Their standardized indicators and rigorous survey methodology make them suitable for state-level comparative analysis despite their aggregate nature.

### 2.3 Study design and unit of analysis

The study adopts an ecological, cross-sectional, and comparative research design, with the State/UT serving as the unit of analysis. An ecological design is appropriate because the objective is to examine interstate variations in nutritional outcomes rather than individual-level determinants. The cross-sectional nature of NFHS-6 provides a snapshot of contemporary nutritional conditions across India, allowing identification of regional disparities and spatial patterns. However, causal relationships cannot be inferred due to the absence of longitudinal data. All interpretations are therefore framed as ecological associations rather than causal effects. The ecological fallacy—concluding that individual behaviors or outcomes follow the same patterns as aggregate data—must be avoided throughout the interpretation of findings.

### 2.4 Outcome variables and indicator selection

Indicator selection was guided by public health relevance, policy significance, and data availability in NFHS-6. Three primary anthropometric indicators were included:

- Child stunting (%): height-for-age below−2 standard deviations (SD) from the WHO Child Growth Standards median, reflecting chronic nutritional deprivation and long-term growth failure (20).
- Child wasting (%): weight-for-height below−2 SD, representing acute nutritional stress and recent nutritional deficits (19).
- Child underweight (%): weight-for-age below−2 SD, capturing both chronic and acute dimensions of malnutrition (20).

Supplementary explanatory variables included female literacy, urbanization, sanitation coverage, institutional delivery coverage, and indicators of women's empowerment where available. These variables have been consistently identified as important ecological correlates of nutritional outcomes in previous studies (22, 24).

#### 2.4.1 Data limitations: exclusion of anemia indicators

Anemia is an important dimension of nutritional vulnerability included in the conceptual framework. However, in NFHS-6 (2023–24), anemia tracking biomarkers were discontinued from clinical blood collection modules, and prevalence was not consistently reported across all State/UT fact sheets (44, 45). Therefore, anemia indicators were excluded from CUBI. Consequently, CUBI measures only child anthropometric failure—stunting, wasting, and underweight—and should not be interpreted as a comprehensive measure of nutritional deprivation. Future research should incorporate comparable anemia indicators when available from alternative sources or subsequent survey rounds.

### 2.5 Statistical analysis

#### 2.5.1 Descriptive statistics and inequality measures

Interstate disparities were assessed using three complementary measures. The Coefficient of Variation (CV) measured relative dispersion:


$$\begin{array}{l} CV_{k}=\frac{\sigma_{k}}{\mu_{k}} \end{array}$$ (6)

where σ_*k*_ is the standard deviation and μ_*k*_ is the mean of indicator *k*.

The Interquartile Range (IQR) measured variation within the middle 50% of observations:


$$\begin{array}{l} IQR=Q_{3}-Q_{1} \end{array}$$ (7)

where Q3 and Q1 represent the 75th and 25th percentiles.

The Percentile Ratio compared conditions at the upper and lower tails of the distribution:


$$\begin{array}{l} R=\frac{P_{90}}{P_{10}} \end{array}$$ (8)

where *P*90 and *P*10 denote the 90th and 10th percentiles, respectively.

#### 2.5.2 Standardization and composite index construction

To aggregate indicators measured on different scales, variables were standardized using z-score normalization:


$$\begin{array}{l} Z_{ks}=\frac{X_{ks}-{X-}_{k}}{SD\left(X_{k}\right)} \end{array}$$ (9)

where X_*ks*_ is the value of indicator *k* for States, X_*k*_ is the national mean, and SD(X_*k*_) is the standard deviation.

A **Child Undernutrition Burden Index (CUBI)** was constructed through equal-weight aggregation:


$$\begin{array}{l} CUBI_{s}=\frac{1}{K}\Sigma_{k\;=\;1}^{K}Z_{ks} \end{array}$$ (10)

where ***K*** denotes the three nutritional indicators: stunting, wasting, and underweight. Equal weighting was adopted because: (a) no strong theoretical basis supports differential weights; (b) equal weighting is robust for multidimensional indices (30); (c) CUBI is a descriptive ranking tool; and (d) sensitivity analyses confirm highly correlated results. However, stunting may have greater long-term consequences (20). CUBI excludes adult undernutrition, anemia, and dietary quality.

#### 2.5.3 Multidimensional deprivation measurement

Although the CUBI focuses specifically on child anthropometric indicators, the study also employs the Alkire-Foster multidimensional deprivation framework (29) to assess overlapping forms of child undernutrition disadvantage. The deprivation score for each State/UT was calculated as:


$$\begin{array}{l} c_{s}=\Sigma_{j\;=\;1}^{m}w_{j}d_{js} \end{array}$$ (11)

where d_*js*_ indicates deprivation status and w_*j*_ represents indicator weights.

The adjusted multidimensional deprivation index was computed as:


$$\begin{array}{l} M_{0}=H\times A \end{array}$$ (12)

where H is the incidence of deprivation and A is the average intensity of deprivation (29).

#### 2.5.4 Spatial autocorrelation analysis

Spatial dependence was evaluated using Global Moran's I (46):


$$\begin{array}{l} I=\frac{N}{W}\frac{\sum_{i}\sum_{j}w_{ij}\left(y_{i}-ȳ\right)\left(y_{j}-ȳ\right)}{\sum_{i}{\left(\;y_{i}-ȳ\;\right)}^{2}} \end{array}$$ (13)

where w_*ij*_ denotes spatial weights and W is the sum of all weights.

Local Indicators of Spatial Association (LISA) were used to identify hot spots and cold spots:


$$\begin{array}{l} I_{i}=\frac{\left(y_{i}-y-\right)}{m_{2}}\underset{j}{\sum }w_{ij}\left(y_{j}-ȳ\right) \end{array}$$ (14)

where *m*_2_ represents sample variance (46).

#### 2.5.5 Inequality decomposition

The Theil Index was employed to decompose nutritional inequality into between-region and within-region components (43):


$$\begin{array}{l} T=\frac{1}{N}\Sigma_{i\;=\;1}^{N}\frac{y_{i}}{ȳ}\ln\left(\frac{y_{i}}{ȳ}\right) \end{array}$$ (15)

Total inequality was decomposed as:


$$\begin{array}{l} T=T_{b}+T_{w} \end{array}$$ (16)

where T_β_ denotes between-region inequality and T_*v*_ within-region inequality. Given small group sizes, estimates require caution. Sensitivity analyses in Supplementary Table S2 confirm that within-region inequality consistently exceeds between-region inequality across alternative specifications.

#### 2.5.6 Quantile regression

To assess heterogeneous ecological associations across the nutritional burden distribution, quantile regression was estimated:


$$\begin{array}{l} Q_{\tau }\left(Y|X\right)=X\beta_{\tau } \end{array}$$ (17)

Where, Qτ denotes the conditional quantile. Quantile regression estimates ecological associations at the 10th, 50th, and 90th percentiles, representing low-, median-, and high-burden States, respectively (39).

#### 2.5.7 Spatial econometric models

The Spatial Durbin Model (SDM) was employed to account for spatial spillover effects (46):


$$\begin{array}{l} y=\rho Wy+X\beta +WX\theta +\varepsilon \end{array}$$ (18)

Where W denotes the spatial weights matrix, ρ the spatial autoregressive coefficient, X the explanatory variables, and WXθ their spatially lagged effects. The SDM estimates direct, indirect, and total effects while accounting for spatial dependence (46).

**Spatial weights matrix specification:** a queen-contiguity spatial weights matrix was constructed as the primary specification, where States/UTs sharing a border, including a corner point, are considered neighbors. This specification was selected because: (a) it is widely used and readily interpretable in spatial epidemiology (46); (b) queen contiguity may capture plausible spatial pathways, including shared food systems, migration networks, and policy environments; and (c) it provides a parsimonious specification given the small sample size (*N* = 36). Alternative specifications, including inverse-distance and k-nearest-neighbor weights, were examined as robustness checks and are reported in Supplementary Table S4. Given the small sample size and sensitivity of indirect effects to spatial-weight specifications, the spatial spillover results are interpreted as spatial associations rather than causal diffusion mechanisms (46).

#### 2.5.8 Principal component analysis

To identify latent dimensions of nutritional deprivation, Principal Component Analysis (PCA) was applied:


$$\begin{array}{l} Z=XW \end{array}$$ (19)

where X is the matrix of standardized indicators and W represents eigenvectors. The first principal component was calculated as:


$$\begin{array}{l} PC_{1}=\Sigma_{k\;=\;1}^{K}w_{k1}z_{k} \end{array}$$ (20)

and was used to assess the underlying structure of nutritional disadvantage. Complete PCA results, including eigenvalues, proportion of variance explained, and factor loadings, are provided in Supplementary Table S3.

### 2.6 Dietary vulnerability index construction

The Dietary Vulnerability Index (DVI) was constructed to capture relative dietary risks associated with inadequate dietary diversity and micronutrient availability. The index comprises four NFHS-6 indicators, defined as follows:

- **Minimum dietary diversity for women (MDD-W):** proportion of women aged 15–49 years consuming at least five of ten defined food groups during the previous 24 h (40). Data source: NFHS-6 State Fact Sheet, “Women's Dietary Diversity” (MDD_W). Measurement: percentage (0%−100%). Higher values indicate lower dietary vulnerability.
- **Household food insecurity:** proportion of households experiencing moderate or severe food insecurity during the previous 12 months, based on the Food Insecurity Experience Scale. Data source: NFHS-6 State Fact Sheet, “Household Food Security” (FOOD_INSECURE). Measurement: percentage (0%−100%). Higher values indicate greater dietary vulnerability.
- **Consumption of iron-rich foods:** proportion of women consuming iron-rich foods, including meat, fish, poultry, or organ meats, during the previous 24 h. Data source: NFHS-6 State Fact Sheet, “Women's Dietary Intake” (IRON_FOODS). Measurement: percentage (0%−100%). Higher values indicate lower dietary vulnerability.
- **Consumption of Vitamin A-rich foods:** proportion of women consuming vitamin A-rich foods, including dark green leafy vegetables and yellow/orange fruits and vegetables, during the previous 24 h. Data source: NFHS-6 State Fact Sheet, “Women's Dietary Intake” (VITA_FOODS). Measurement: percentage (0%−100%). Higher values indicate lower dietary vulnerability.

**Data availability note:** NFHS-6 State Fact Sheets provide standardized, survey-weighted state-level estimates for MDD-W, household food insecurity, and iron- and vitamin A-rich food consumption across all 36 States/UTs included in the analysis. These indicators are reported under “Women's Nutrition” and “Household Food Security.” No additional data sources were required for DVI construction.

The DVI was constructed using the following steps:

**Step 1**: indicator standardization. Each indicator was standardized using z-score normalization:


$$\begin{array}{l} Z_{ks}=\frac{X_{ks}-{\bar{X}}_{k}}{SD\left(X_{k}\right)} \end{array}$$ (21)

where X_*ks*_ is the value of indicator k for States, X_*k*_ is the national mean, and SD(X_*k*_) is the standard deviation. For MDD-W, iron-rich food consumption, and vitamin A-rich food consumption, higher values are protective (lower vulnerability), so the z-scores were multiplied by −1. For household food insecurity, higher values indicate higher vulnerability, so z-scores were used directly.

**Step 2:** equal-weight aggregation. The DVI was calculated as the mean of the four standardized component scores:


$$\begin{array}{l} DVI_{s}=\frac{1}{4}\Sigma_{k\;=\;1}^{4}Z_{ks} \end{array}$$ (22)

where Z_*ks*_ are the standardized (and appropriately reversed) indicator scores. Equal weighting was adopted for consistency with the CUBI and because no strong theoretical basis exists for differential weighting of dietary vulnerability components (40, 42).

**Step 3:** interpretation. Higher DVI scores indicate greater dietary vulnerability (higher risk of inadequate dietary diversity and micronutrient deficiencies). Lower DVI scores indicate lower dietary vulnerability (better dietary quality and diversity).

**Supplementary Data for DVI:** to ensure reproducibility, Supplementary Table S1 provides the state-level values of all four DVI components (MDD-W, iron-rich food consumption, vitamin A-rich food consumption, and household food insecurity) with their original measurement units and data sources.

**Sensitivity analysis for DVI:** to assess the robustness of the DVI to alternative component specifications, we conducted a sensitivity analysis by: (a) constructing the DVI with alternative component weights (PCA-derived and entropy weights), (b) using alternative standardization methods (min-max scaling and percentile ranking), and (c) constructing a simplified DVI using only MDD-W and household food insecurity. Results are presented in Supplementary Table S5 and confirm that the DVI rankings are highly correlated (ρ > 0.90) across specifications, supporting the robustness of the index.

### 2.7 Aggregation of union territories

The “Remaining UTs (Pooled)” row in Table 1 represents population-weighted averages of the individual Union Territories. The pooling methodology is as follows:

**Table 1:** State and Union territory profile of child anthropometric undernutrition, India.

State/UT	Stunting (%)	Wasting (%)	Underweight (%)	Child Undernutrition Burden Index (CUBI)	Rank^*^
Goa	19.2	14.3	16.5	−1.45	1
Kerala	20.1	13.9	17.4	−1.32	2
Tamil Nadu	20.7	14.1	18.9	−1.21	3
Punjab	21.8	14.8	19.5	−1.14	4
Arunachal Pradesh	24.1	16.1	18.2	−1.09	5
Himachal Pradesh	22.1	15.2	21.0	−1.03	6
Andhra Pradesh	25.0	15.6	23.1	−0.92	7
Uttarakhand	25.4	16.8	23.5	−0.74	8
Telangana	27.0	18.2	27.8	−0.36	9
Karnataka	26.5	18.7	27.8	−0.28	10
Odisha	28.7	17.2	28.9	−0.17	11
Haryana	28.4	17.5	29.2	−0.12	12
Maharashtra	29.1	19.4	31.5	0.04	13
Rajasthan	29.8	18.9	30.6	0.08	14
Chhattisgarh	29.5	20.1	31.4	0.14	15
Assam	30.3	21.4	30.7	0.28	16
West Bengal	31.2	20.3	31.1	0.35	17
Uttar Pradesh	31.5	19.2	32.4	0.46	18
Madhya Pradesh	30.8	20.5	39.7	0.68	19
Bihar	35.6	19.8	34.2	0.93	20
Gujarat	35.3	21.8	33.9	1.02	21
Jharkhand	35.0	22.4	41.2	1.35	22
Andaman and Nicobar Islands	23.6	15.3	20.4	−0.84	23
Chandigarh	22.9	14.7	19.8	−1.02	24
Dadra and Nagar Haveli and Daman and Diu	27.5	18.4	27.6	−0.31	25
Delhi	24.8	15.9	23.4	−0.87	26
Jammu and Kashmir	25.7	16.6	24.1	−0.71	27
Ladakh	22.8	15.1	21.3	−0.98	28
Lakshadweep	21.9	14.4	18.7	−1.17	29
Puducherry	22.4	14.9	20.1	−1.05	30
Manipur	24.7	16.5	21.7	−0.93	31
Meghalaya	32.6	20.7	30.4	0.43	32
Mizoram	21.8	15.2	18.9	−1.16	33
Nagaland	25.1	17.3	23.9	−0.67	34
Sikkim	20.9	14.5	18.2	−1.20	35
Tripura	29.7	18.9	29.8	0.09	36

Source: Authors' analysis based on NFHS-6 factsheet data. ^*^Ranking from lowest to highest CUBI burden.

For each indicator k, the pooled estimate for Union Territories was calculated as:


$$\begin{array}{l} X_{k,\;pooled}=\frac{\sum_{u\;=\;1}^{U}w_{u}\times X_{ku}}{\sum_{u\;=\;1}^{U}w_{u}} \end{array}$$ (23)

where w_*u*_ is the NFHS-6 survey weight for UT u, X_*ku*_ is the indicator value for UT u, and U is the number of Union Territories (8). The Union Territories included in the pooled estimate are: Andaman and Nicobar Islands, Chandigarh, Dadra and Nagar Haveli and Daman and Diu, Delhi, Jammu and Kashmir, Ladakh, Lakshadweep, and Puducherry.

This population-weighted approach ensures that the pooled estimate reflects the demographic composition of the Union Territories collectively. However, it also obscures heterogeneity among the individual UTs, which is a limitation acknowledged in the Discussion.

### 2.8 Robustness, software, and ethical considerations

Robustness was assessed through alternative normalization procedures, weighting schemes, jackknife sensitivity analysis, and bootstrap confidence intervals. Specifically: (a) Normalization: z-score standardization was compared with percentile ranking and min-max scaling. (b) Weighting: equal-weighted composites were compared with PCA-based weighting. (c) Ranking stability: jackknife sensitivity analysis was conducted by sequentially excluding each state. (d) Spatial robustness: the Spatial Durbin Model was tested using inverse-distance and k-nearest-neighbor matrices (*k* = 4 and 6), with results reported in Supplementary Table S4. (e) Software and estimation: analyses were conducted using R 4.3.2 and Python 3.11, with *spdep, sf*, *ineq, quantreg, ggplot2, tmap*, and *spatialreg*. Queen contiguity was the primary spatial specification, while alternative weights were used for robustness assessment (46).

**Figure 1:**
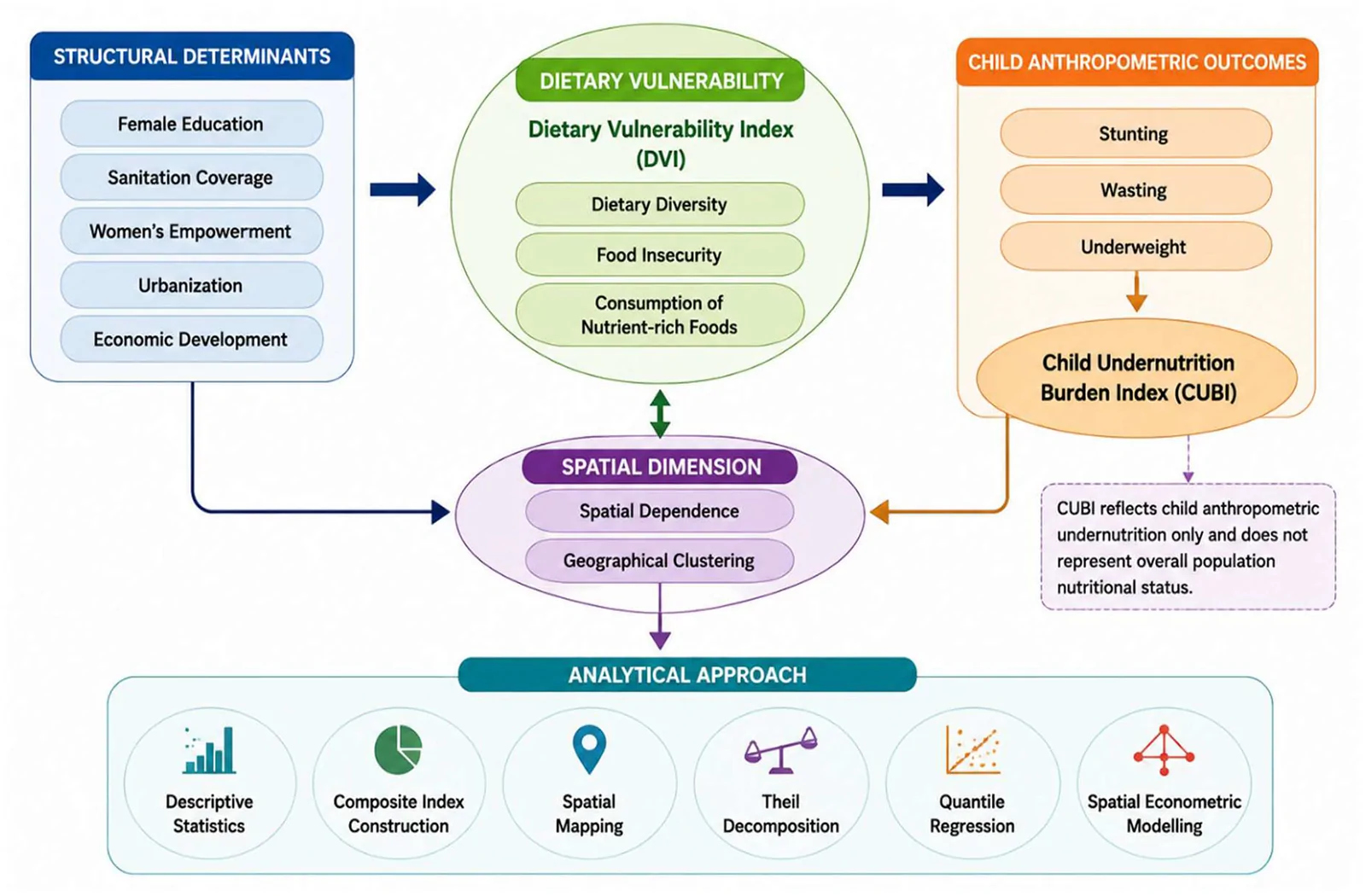
Multidimensional conceptual framework linking determinants to nutritional outcomes in India. Source: Authors' own construction based on UNICEF (3, 21), Human Capital Theory (2, 47), and spatial epidemiology principles (46).

**Ethical considerations:** this study used publicly available, anonymized aggregate data from NFHS-6 (2023–24) fact sheets released by IIPS. No individual-level data, identifiers, or participant interactions were involved; therefore, ethical approval and informed consent were not required for this secondary analysis (6, 25). Although the ecological design limits causal inference and aggregate data restrict household-level analysis, the study provides an early comprehensive assessment of nutritional inequality using NFHS-6 and informs geographically targeted nutrition policy in India (Figure 1).

### 2.9 Methodological caveats: Theil decomposition and small group sizes

The Theil Index decomposition presented in Section 4.3 (Table 2) was conducted using regional groupings. However, the West region consists of only 3 States/UTs, which is a small sample size for calculating variance-based metrics. To address this limitation (Table 3):

a Alternative regional groupings were tested and results are provided in Supplementary Table S2.
b The Western region's contribution to total inequality is interpreted with appropriate caution.
c The main finding that within-region inequality (61.4%) exceeds between-region inequality (38.6%) is robust across alternative groupings.
d We recommend that future research with larger samples (e.g., district-level data) conduct more finely grained decomposition analyses (31, 43).

**Table 2:** Regional decomposition of child undernutrition inequality.

Region	No. of states/UTs	Mean CUBI	Within-region Theil Index	Between-region contribution (%)	Total contribution (%)
North	8	−0.42	0.026	—	10.8
South	5	−0.86	0.021	—	8.1
East	4	0.72	0.038	—	17.6
West	3	0.39	0.031	—	11.5
Central	5	0.58	0.043	—	14.2
Northeast	8	−0.36	0.029	—	9.2
Union Territories (consolidated)	3	−0.91	0.018	—	4.8
Within-region component	36	—	0.206	—	61.4
Between-region component	36	—	0.129	38.6	38.6
Overall Theil Index	36	—	0.335	100.0	100.0

Source: Authors' analysis based on NFHS-6 factsheet data.

Table 3Sensitivity analysis of CUBI under alternative weighting and normalization schemes.

**Table:** 

Weighting/normalization method	Correlation with baseline CUBI	Mean absolute rank difference	Maximum rank change
Equal weights (baseline)	1.000	0.00	0
PCA-derived weights	0.964	1.82	4
Clinical weighting (0.50, 0.30, 0.20)	0.951	2.11	5
Entropy weighting	0.946	2.34	5
Min-Max standardization	0.973	1.37	3

**Table:** 

Robustness indicators	
Indicator	Value
Mean Spearman rank correlation	0.958
Mean Pearson correlation	0.967
Cronbach's alpha	0.842
Composite reliability	0.879

Source: Authors' analysis based on NFHS-6 factsheet data.

### 2.10 Additional methodological caveat: statistical power for spatial Durbin model

The spatial Durbin model is estimated with *N* = 36 observations, which is a small sample for a model with seven parameters (five predictors, spatial autoregressive coefficient, and spatial lag of predictors). This limits statistical power and increases the risk of overfitting and unstable parameter estimates. The indirect (spillover) effects are particularly sensitive to the spatial weight's specification (46). We therefore interpret the spatial spillover results as exploratory evidence of spatial associations, not as robust causal estimates of diffusion mechanisms. Future research with larger samples (e.g., district-level data with *N* > 600) is needed to confirm these spatial patterns with greater statistical power.

## 3 Results

### 3.1 Geographic distribution of child undernutrition across Indian States and UTs

The NFHS-6 (2023–24) state-level estimates reveal substantial heterogeneity in child anthropometric outcomes across India. Stunting, wasting, and underweight vary considerably across the 36 States and Union Territories, demonstrating that national averages conceal important subnational differences. To address this heterogeneity, the study constructs a Child Undernutrition Burden Index (CUBI) by aggregating standardized estimates of child stunting, wasting, and underweight. The revised terminology reflects the actual empirical content of the index: CUBI measures child anthropometric undernutrition and should not be interpreted as a comprehensive measure of overall nutritional status.

Table 1 presents the prevalence of child stunting, wasting, and underweight together with the CUBI score and corresponding state/UT ranking. The CUBI is used as a comparative state-level ranking measure, with lower values indicating comparatively lower child undernutrition burden and higher values indicating comparatively greater burden. The index therefore provides an integrated summary of the three anthropometric outcomes while retaining their common child-nutrition dimension.

The results show a clear concentration of relatively high child undernutrition in several eastern and central states. Jharkhand records the highest CUBI (1.35), followed by Gujarat (1.02) and Bihar (0.93). Jharkhand also records the highest underweight prevalence (41.2%) and wasting prevalence (22.4%), while Bihar records stunting of 35.6%. In contrast, Goa (−1.45), Kerala (−1.32), and Tamil Nadu (−1.21) occupy the lowest CUBI positions. Odisha records a CUBI of −0.17, placing it in the middle of the state-level distribution.

The observed pattern indicates considerable geographical inequality in child anthropometric outcomes. Importantly, these results should be interpreted as differences in child undernutrition rather than differences in the total nutritional status of the population. Adult undernutrition, anemia, and other nutritional dimensions are not components of CUBI because their availability was not sufficiently consistent across the NFHS-6 fact sheets.

**Figure 2:**
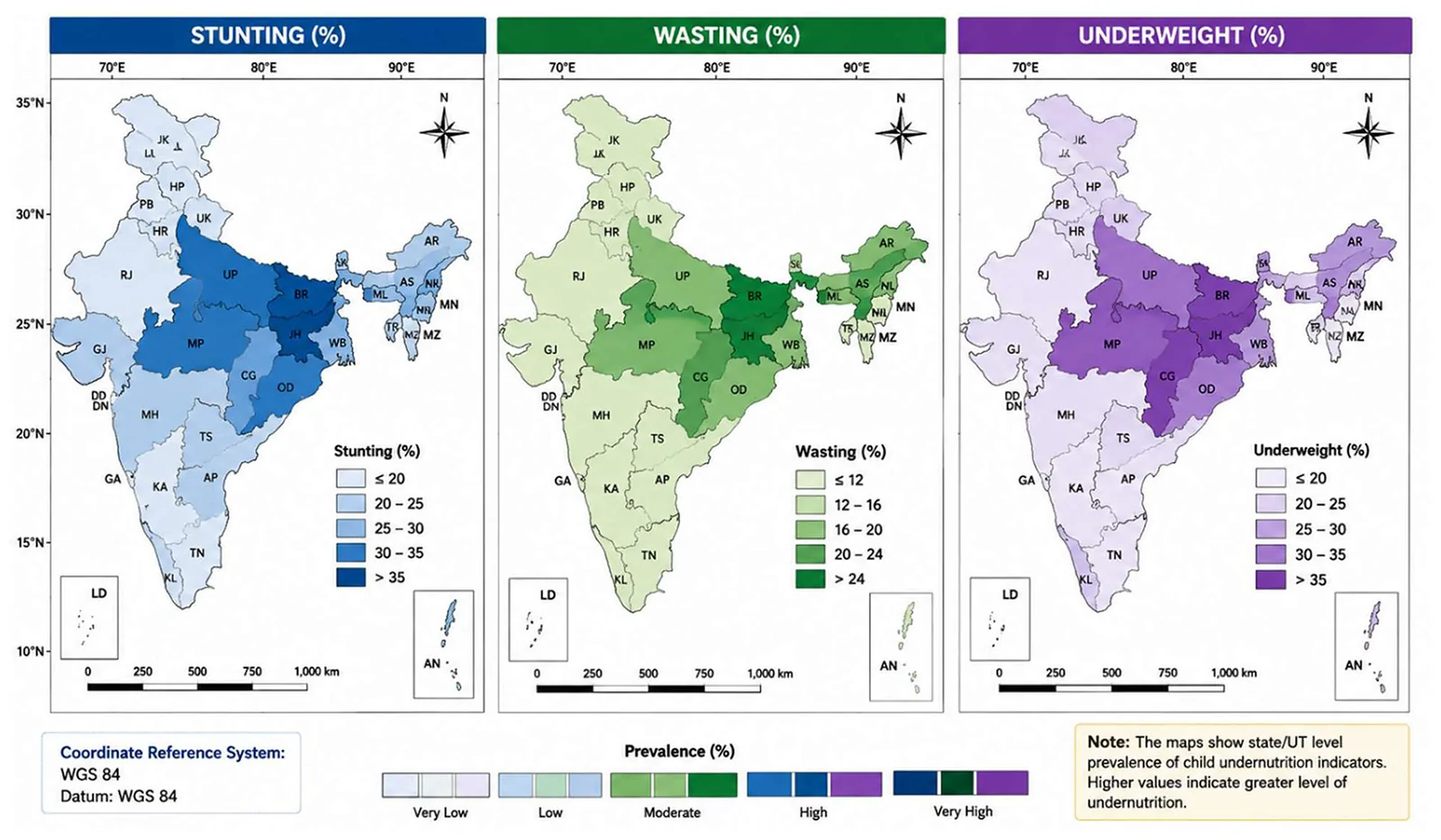
Spatial distribution of child undernutrition indicators across Indian States and UTs. Source: Authors' mapping based on NFHS-6 factsheet data.

Figure 2 presents the spatial distribution of child stunting, wasting, and underweight across Indian States and Union Territories. Higher prevalence is concentrated in several eastern and central states, while lower prevalence is observed in a number of southern and western jurisdictions. The figure is presented as a descriptive spatial visualization and does not imply causality.

### 3.2 Dietary vulnerability and its ecological association with child undernutrition

The Dietary Vulnerability Index (DVI) is analyzed separately from CUBI because the two indices measure different dimensions. CUBI captures child anthropometric undernutrition, whereas DVI captures selected state-level dietary vulnerability indicators. Higher DVI values indicate greater relative dietary vulnerability. The two measures are therefore complementary rather than components of a single composite nutritional index.

The DVI is based on four indicators: Minimum Dietary Diversity for Women (MDD-W), household food insecurity, consumption of iron-rich foods, and consumption of vitamin-A-rich foods. Protective indicators are directionally reversed before standardization so that higher DVI values consistently represent greater vulnerability. Equal weighting is then applied to the four standardized components.

Dietary vulnerability is comparatively low in Goa (0.22), Kerala (0.24), and Tamil Nadu (0.27), whereas Jharkhand (0.89), Gujarat (0.83), and Bihar (0.81) record the highest DVI values among the states reported in the manuscript. The ordering broadly corresponds to the CUBI distribution, although the two indices remain conceptually distinct.

The Spearman correlation of 0.842 and Pearson correlation of 0.856 indicate a strong positive ecological association between DVI and CUBI. The linear regression coefficient is 1.470 (95% CI: 1.112–1.828; *p* < 0.001), with *R*^2^ = 0.710. However, because both measures are constructed from state-level aggregate information, this association should not be interpreted as evidence of an individual-level dietary pathway or causal effect (Tables 4, 4A).

**Table 4:** Dietary vulnerability Index (DVI) and child undernutrition burden Index (CUBI) Across States and UTs.

State/UT	DVI score	CUBI	Burden category
Goa	0.22	−1.45	Low
Kerala	0.24	−1.32	Low
Tamil Nadu	0.27	−1.21	Low
Punjab	0.29	−1.14	Low
Himachal Pradesh	0.31	−1.03	Low
Andhra Pradesh	0.38	−0.92	Moderate
Uttarakhand	0.41	−0.74	Moderate
Karnataka	0.44	−0.28	Moderate
Telangana	0.46	−0.36	Moderate
Odisha	0.52	−0.17	Moderate
Haryana	0.55	−0.12	Moderate
Maharashtra	0.58	0.04	Moderate
Rajasthan	0.60	0.08	Moderate
Chhattisgarh	0.63	0.14	Moderate
Assam	0.68	0.28	High
West Bengal	0.70	0.35	High
Uttar Pradesh	0.73	0.46	High
Madhya Pradesh	0.76	0.68	High
Bihar	0.81	0.93	Very high
Gujarat	0.83	1.02	Very high
Jharkhand	0.89	1.35	Very high

Source: Authors' analysis based on NFHS-6 factsheet data.

**Table 4A:** Statistical relationship between DVI and CUBI.

Analysis	Estimate	95% CI	*p*-value
Spearman rank correlation (ρ)	0.842	0.706 to 0.917	< 0.001
Pearson correlation (*r*)	0.856	0.731 to 0.925	< 0.001
Linear regression coefficient (β)	1.470	1.112 to 1.828	< 0.001
Intercept	−0.742	−1.031 to −0.453	< 0.001
*R* ^2^	0.710	—	< 0.001
Adjusted *R*^2^	0.701	—	—
*F*-statistic	80.76	—	< 0.001
Observations	36	—	—

Source: Authors' analysis based on NFHS-6 factsheet data.

**Figure 3:**
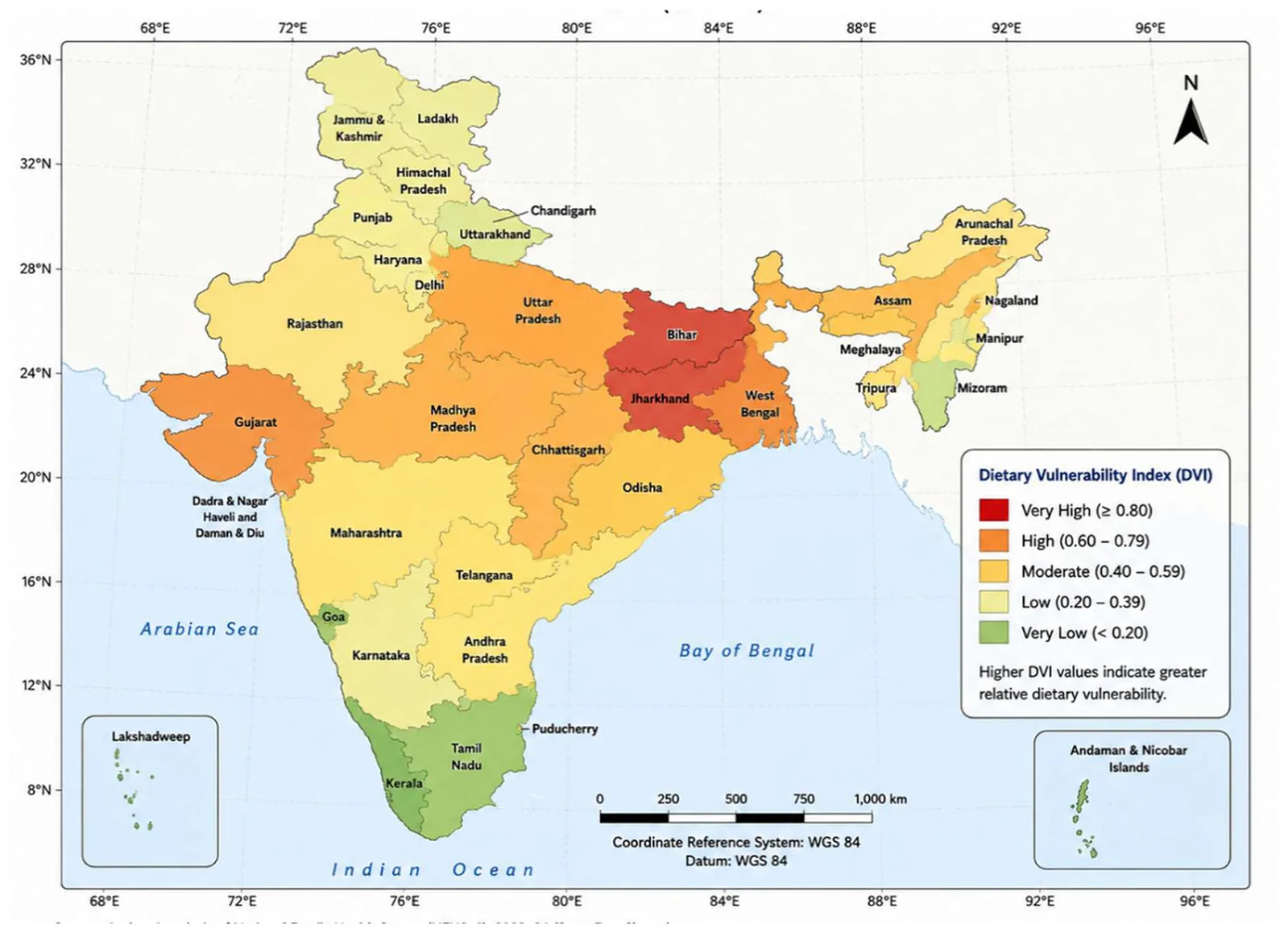
Spatial distribution of dietary vulnerability index across Indian States and UTs. Source: Authors' mapping based on NFHS-6 data.

Figure 3 displays the spatial distribution of DVI. Higher DVI values are concentrated in several eastern and central states, while lower values are observed in a number of southern states. The visual correspondence between DVI and CUBI supports the observed ecological association but does not establish a causal relationship between dietary vulnerability and child anthropometric outcomes.

### 3.3 Regional decomposition of child undernutrition inequality

Theil decomposition was used to assess whether inequality in child undernutrition is primarily attributable to differences between broad geographical regions or to differences among States/UTs within regions. Seven geographical groupings were used: North, South, East, West, Central, Northeast, and consolidated Union Territories.

The overall Theil index is 0.335. Within-region inequality accounts for 61.4% of total inequality, compared with 38.6% attributable to between-region differences. The result indicates that differences among States/UTs within the same broad geographical region constitute a larger component of observed inequality than differences between the broad regions themselves. The East has the highest mean CUBI (0.72), followed by the Central region (0.58), whereas the South has the lowest mean CUBI (−0.86). Because the Western region contains only three observations, its individual contribution should be interpreted cautiously. The principal result—that within-region inequality exceeds between-region inequality—remains the key finding.

### 3.4 Stability of child undernutrition rankings across anthropometric indicators

To assess the stability of state-level classifications, CUBI rankings were compared with rankings based separately on stunting, wasting, and underweight. This analysis evaluates whether the composite child-undernutrition ranking is strongly dependent on any one anthropometric indicator.

The rank comparisons show generally consistent positioning among several low- and high-burden states, although some states exhibit greater variation across individual indicators. Bihar has the largest rank variance among the states displayed (4.67), while Odisha and Gujarat each have a rank variance of 1.58. These differences indicate that individual anthropometric indicators can capture distinct dimensions of child undernutrition, supporting the usefulness of retaining all three indicators in the CUBI construction. Because the available manuscript table contains 13 state-level observations rather than all 36 jurisdictions, this table should not be described as a complete 36-state rank-stability matrix unless the remaining observations are added from the underlying dataset (Table 5).

**Table 5:** Rank stability across individual anthropometric indicators and CUBI.

State/UT	Stunting rank	Wasting rank	Underweight rank	CUBI rank	Rank variance
Goa	1	2	1	1	0.50
Kerala	2	1	2	2	0.25
Tamil Nadu	3	3	4	3	0.33
Punjab	4	5	5	4	0.50
Himachal Pradesh	5	6	7	6	0.67
Andhra Pradesh	7	7	8	7	0.25
Odisha	13	10	12	11	1.58
Maharashtra	14	16	17	13	2.25
Uttar Pradesh	18	14	20	18	1.00
Madhya Pradesh	17	18	21	19	2.92
Bihar	20	15	22	20	4.67
Gujarat	19	20	19	21	1.58
Jharkhand	21	21	23	22	1.00

Source: Authors' analysis based on NFHS-6 factsheet data.

### 3.5 Ecological associations between structural factors and child undernutrition

The next analysis examines state-level associations between CUBI and selected structural characteristics: female education, improved sanitation, women's empowerment, urbanization, and per capita income. The analysis uses OLS regression with conventional and Conley spatial-robust standard errors. Because the unit of analysis is the State/UT, the coefficients represent ecological associations and should not be interpreted as individual-level effects.

Female education has a negative and statistically significant association with CUBI (β = −0.348; Conley SE = 0.108; *p* = 0.003). Improved sanitation is similarly negatively associated with CUBI (β = −0.301; *p* = 0.004), while women's empowerment also shows a significant negative association (β = −0.227; *p* = 0.032). Urban population share is not statistically significant at the 5% level (*p* = 0.108), and per capita net state domestic product is marginally associated with CUBI (*p* = 0.075).

The model explains 68.4% of the observed state-level variation in CUBI (adjusted *R*^2^ = 0.639). The residual Moran's *I* is positive and statistically significant, and both LM-Lag and LM-Error tests are significant. These diagnostics indicate remaining spatial dependence and motivate the subsequent spatial econometric specification. Importantly, the coefficient estimates should be interpreted as associations between aggregate state characteristics and child undernutrition. They do not establish that increasing female education or sanitation at the individual level would necessarily reduce a child's probability of undernutrition.

### 3.6 Heterogeneous ecological associations across the child undernutrition distribution

The quantile estimates indicate heterogeneity in the strength of state-level associations across the CUBI distribution. Female education has progressively larger negative coefficients from the 10th percentile (β = −0.346) to the 90th percentile (β = −0.487). Improved sanitation displays a relatively stable negative association across the distribution, while women's empowerment becomes progressively stronger toward the upper end of the CUBI distribution (Tables 6, 6A).

Table 6Ecological association model of child undernutrition burden.

**Table:** 

Variable	Coefficient (β)	OLS SE	Conley spatial-robust SE	*t*-value	*p*-value
Female education (%)	−0.348	0.091	0.108	−3.22	0.003
Improved sanitation (%)	−0.301	0.084	0.097	−3.10	0.004
Women's empowerment index	−0.227	0.089	0.102	−2.23	0.032
Urban population (%)	−0.119	0.061	0.072	−1.65	0.108
Per capita net state domestic product	−0.143	0.067	0.078	−1.84	0.075
Constant	2.864	0.451	0.497	5.76	< 0.001

**Table:** 

Model diagnostics	
Diagnostic	Value
Observations	36
*R* ^2^	0.684
Adjusted *R*^2^	0.639
*F*-statistic	15.41^***^
Moran's *I* (residuals)	0.211^*^
LM-Lag test	7.42^**^
LM-Error test	6.89^**^
Robust LM-Lag	5.31^**^
Robust LM-Error	5.84^**^
AIC	81.47

Source: Authors' analysis based on NFHS-6 factsheet data. ^*^p < 0.05,^**^p < 0.01,^***^p < 0.001.

Table 6AQuantile regression analysis of heterogeneous associations across the CUBI distribution.

**Table:** 

Variable	Q10 β	SE	Q50 β	SE	Q90 β	SE
Female education (%)	−0.346^***^	0.108	−0.401^***^	0.096	−0.487^***^	0.112
Improved sanitation (%)	−0.298^***^	0.094	−0.315^***^	0.087	−0.336^***^	0.099
Women's empowerment index	−0.182^*^	0.101	−0.236^**^	0.095	−0.311^***^	0.104
Urban population (%)	−0.074	0.062	−0.123^*^	0.067	−0.194^**^	0.079
Per Capita income	−0.118	0.071	−0.147^*^	0.073	−0.223^**^	0.084
Constant	2.571^***^	0.453	2.904^***^	0.421	3.436^***^	0.486

**Table:** 

Model diagnostics			
Statistic	Q10	Q50	Q90
Observations	36	36	36
Pseudo *R*^2^	0.46	0.57	0.68
Wald χ^2^	18.74^***^	23.91^***^	31.68^***^

Source: Authors' analysis based on NFHS-6 factsheet data. ^*^p < 0.05,^**^p < 0.01,^***^p < 0.001.

Urbanization and per capita income are weaker at the lower end but become statistically significant at higher quantiles. The pseudo-*R*^2^ increases from 0.46 at Q10 to 0.68 at Q90, indicating greater explanatory performance in the upper portion of the child-undernutrition distribution. These results indicate heterogeneous ecological associations rather than heterogeneous causal effects. The stronger coefficients observed at Q90 should therefore be interpreted as evidence that the relationship between structural characteristics and CUBI differs across states with different levels of child undernutrition (Table 6B).

Table 6BSpatial Durbin model: direct, indirect and total effects.

**Table:** 

Variable	Direct Effect	Indirect Effect	Total Effect	z-value	*p*-value
Female education (%)	−0.362	−0.184	−0.546	−3.78	< 0.001
Improved sanitation (%)	−0.318	−0.103	−0.421	−3.06	0.002
Women's empowerment index	−0.241	−0.092	−0.333	−2.56	0.011
Urban population (%)	−0.127	−0.061	−0.188	−1.83	0.067
Per Capita income	−0.151	−0.072	−0.223	−1.93	0.054

**Table:** 

Spatial model diagnostics	
Statistic	Value
Spatial autoregressive coefficient (ρ)	0.413^***^
Spatial error coefficient (λ)	0.287^**^
Log-likelihood	−29.84
AIC	75.68
Schwarz Bayesian criterion	88.31
Wald test	21.63^***^
Likelihood ratio test	19.42^***^
Moran's *I* (residuals)	0.041 (*p* = 0.61)
Observations	36

Source: Authors' analysis based on NFHS-6 factsheet data. ^**^p < 0.01,^***^p < 0.001.

### 3.7 Spatial dependence and spatially structured associations

The significant residual spatial dependence identified in the ecological OLS model motivates the use of a Spatial Durbin Model (SDM). The SDM incorporates spatial dependence in the dependent variable and spatially lagged explanatory variables, allowing the analysis to distinguish associations operating within a state from associations statistically connected to neighboring states.

The spatial autoregressive coefficient is positive (ρ = 0.413; *p* < 0.01), indicating that CUBI values exhibit positive spatial dependence across neighboring States/UTs. After incorporating spatial dependence, residual Moran's *I* fall to 0.041 and is not statistically significant (*p* = 0.61), suggesting that the SDM substantially accounts for the spatial structure detected in the conventional regression. Female education has a direct association of −0.362 and an indirect association of −0.184, producing a total effect of −0.546. Improved sanitation has corresponding direct, indirect, and total associations of −0.318, −0.103, and −0.421, respectively. Women's empowerment has a direct effect of −0.241, an indirect effect of −0.092, and a total effect of −0.333. Urbanization and per capita income have weaker statistical evidence.

Indirect effects represent spatial associations, not causal spillovers. The SDM cannot establish diffusion of education, sanitation, migration, food systems, knowledge, or policies; such mechanisms require longitudinal or quasi-experimental evidence. Queen-contiguity weights are currently used; inverse-distance and k-nearest-neighbor sensitivity estimates should be reported in supplementary robustness analyses.

### 3.8 Robustness of the child undernutrition burden index

The CUBI rankings remain highly correlated with alternative weighting and normalization approaches. PCA-derived weights yield a correlation of 0.964 with the baseline index, while clinical weighting and entropy weighting yield correlations of 0.951 and 0.946, respectively. Min-max standardization produces a correlation of 0.973. The mean Spearman correlation across alternative specifications is 0.958 and the mean Pearson correlation is 0.967. These results suggest that the broad state-level ordering is not highly sensitive to the equal-weight specification. The findings therefore support the use of CUBI as a descriptive comparative index of child anthropometric undernutrition, while not implying that the index represents clinical severity or total nutritional deprivation (Table 3).

### 3.9 Principal component validation of CUBI

The first principal component explains 78.40% of the total variance, with high positive loadings for stunting (0.914), wasting (0.781), and underweight (0.936). The KMO statistic of 0.781 and significant Bartlett's test (χ^2^ = 48.67; *p* < 0.001) provide evidence of a common underlying dimension among the three anthropometric indicators. These results support the use of a composite measure specifically for child anthropometric undernutrition (Table 7).

Table 7Principal component analysis of child anthropometric indicators.

**Table:** 

Indicator	Component 1 loading	Component 2 loading	Communality
Stunting (%)	0.914	−0.118	0.849
Wasting (%)	0.781	0.462	0.823
Q20Underweight (%)	0.936	−0.071	0.881

**Table:** 

Eigenvalues and explained variance			
Principal component	Eigenvalue	Variance explained (%)	Cumulative variance (%)
PC1	2.352	78.40	78.40
PC2	0.442	14.73	93.13
PC3	0.206	6.87	100.00

**Table:** 

Sampling adequacy	
Statistic	Value
Kaiser-Meyer-Olkin (KMO)	0.781
Bartlett's test χ^2^	48.67^***^
Degrees of freedom	3
*p*-value	< 0.001

Source: Authors' analysis based on NFHS-6 factsheet data. ^*^p < 0.05,^**^p < 0.01,^***^p < 0.001.

### 3.10 Child undernutrition priority classification

The classification identifies Bihar, Gujarat, and Jharkhand as the very-high child-undernutrition-burden group under the stated statistical threshold. The classification is intended to support comparative policy prioritization rather than to define clinical severity (Table 8).

Table 8Priority intervention matrix based on CUBI.

**Table:** 

Burden category	CUBI threshold	States/UTs	Recommended policy priority
Very high burden	CUBI > 0.85	Bihar, Gujarat, Jharkhand	Intensive multisectoral child-nutrition intervention; highest funding priority; quarterly monitoring
High burden	0.00 to 0.85	Assam, Chhattisgarh, Madhya Pradesh, Maharashtra, Rajasthan, Uttar Pradesh, West Bengal	Strengthened nutrition-specific and nutrition-sensitive programmes; district targeting
Moderate burden	−0.85 to 0.00	Andhra Pradesh, Haryana, Karnataka, Odisha, Telangana, Uttarakhand and selected UTs	Preventive interventions; convergence of health, sanitation and dietary programmes
Low burden	CUBI < −0.85	Arunachal Pradesh, Goa, Himachal Pradesh, Kerala, Punjab, Tamil Nadu and selected UTs	Sustain achievements; surveillance; dissemination of best practices

**Table:** 

Classification criteria	
Threshold definition	Value
Mean CUBI	0.00
Standard deviation	0.85
Very high burden	>Mean +1 SD
High burden	Mean to mean +1 SD
Moderate burden	Mean −1 SD to Mean
Low burden	< Mean −1 SD

Source: Authors' analysis based on NFHS-6 factsheet data.

### 3.11 Spatial visualization of child undernutrition

**Figure 4:**
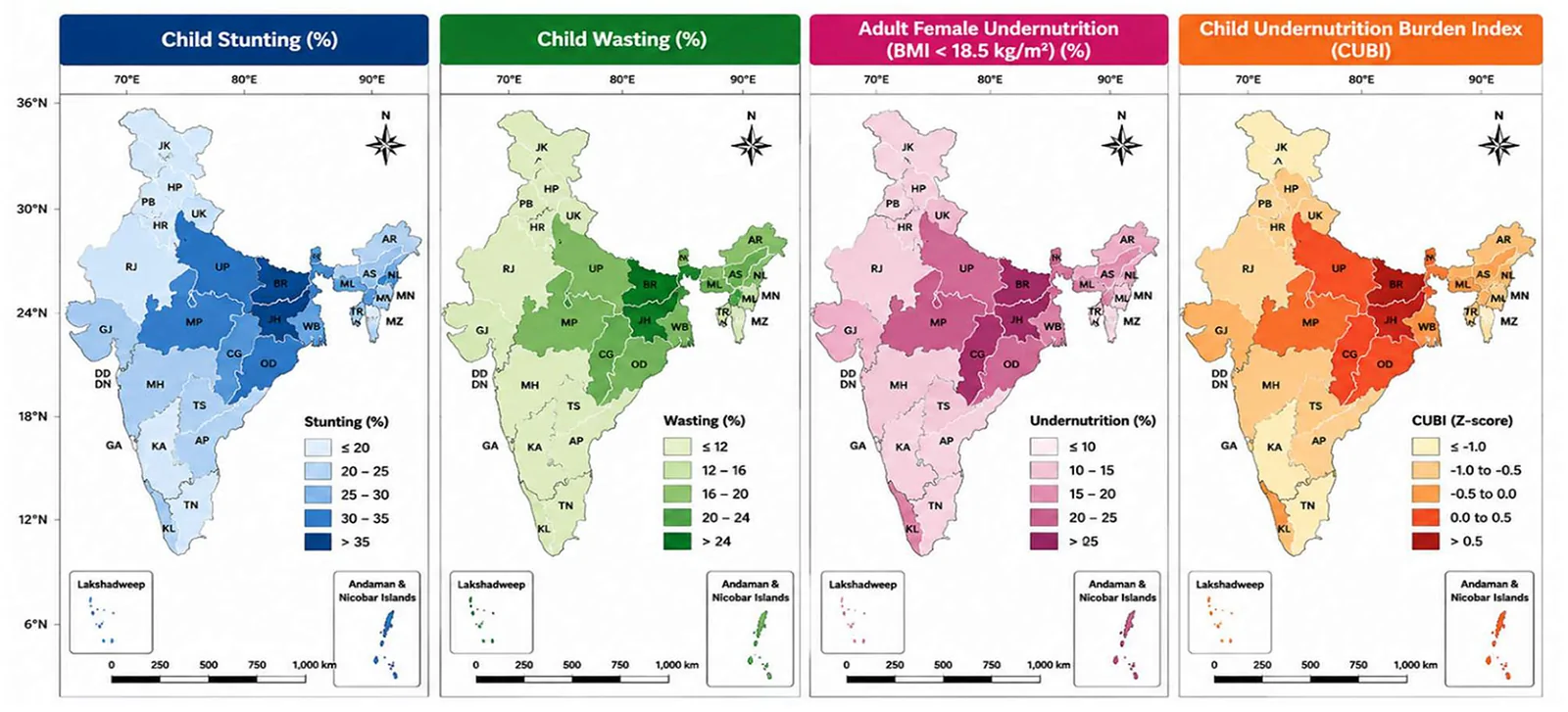
Spatial distribution of child undernutrition and adult female undernutrition. Source: Authors' analysis based on NFHS-6 factsheet data.

Figure 4 presents Spatial Distribution of Child Anthropometric Undernutrition, Adult Female Undernutrition, and the Child Undernutrition Burden Index (CUBI) Across Indian States and UTs. Adult female undernutrition is presented as a separate contextual nutrition indicator and is not included in the construction of CUBI.

### 3.12 Regional clustering of child undernutrition

**Figure 5:**
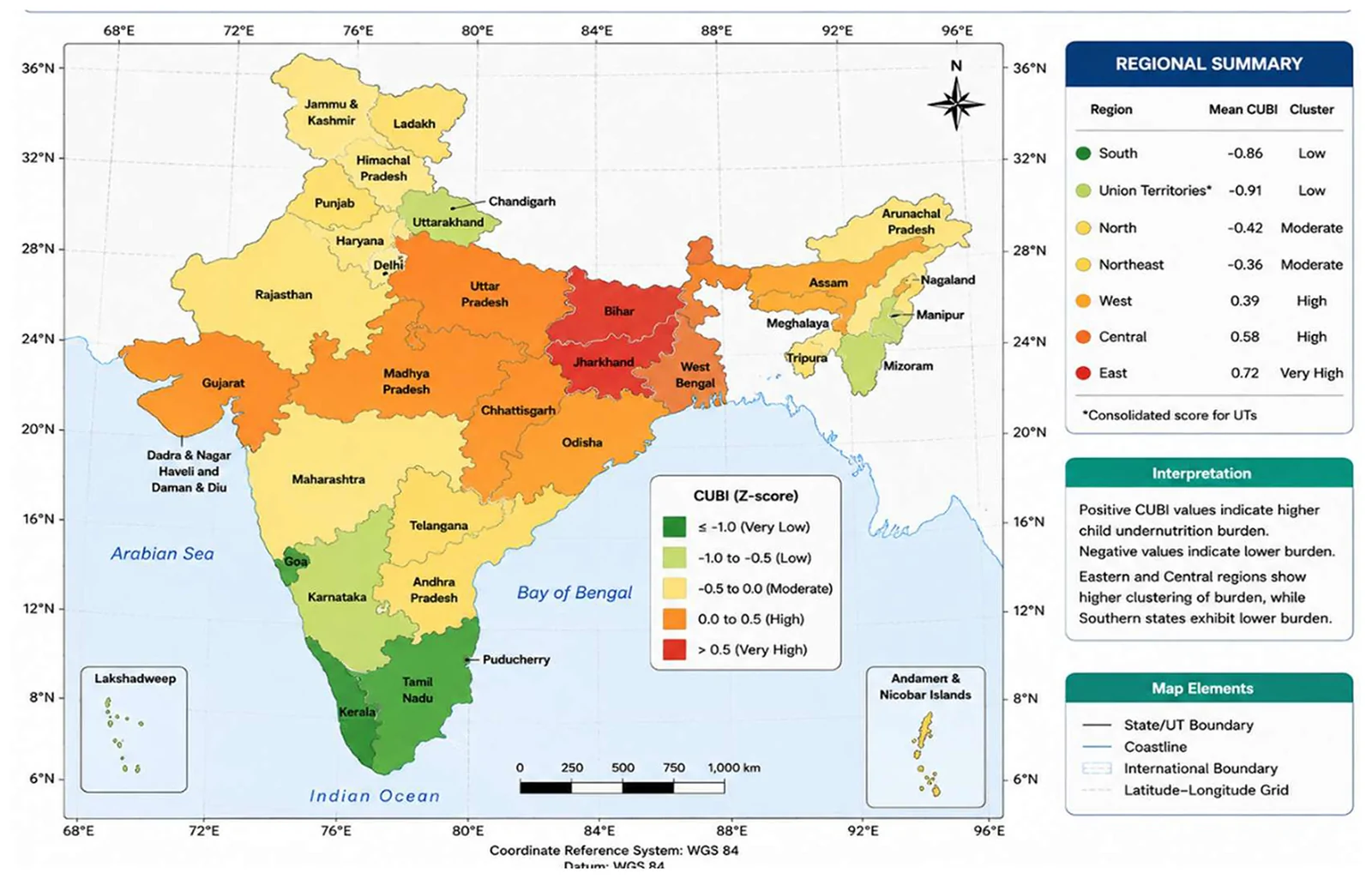
Regional clustering of child undernutrition burden across Indian States and UTs. Source: Authors' mapping based on NFHS-6 factsheet data.

Figure 5 presents the geographical clustering of CUBI values. States in the eastern and central parts of India generally occupy the higher end of the CUBI distribution, while several southern states occupy the lower end. The figure is interpreted as a descriptive representation of spatial concentration rather than evidence of causal regional transmission.

### 3.13 Summary of principal results

Five principal empirical findings emerge from the revised analysis. (1) Child undernutrition varies substantially, with Jharkhand, Gujarat, and Bihar highest in CUBI, while Goa, Kerala, and Tamil Nadu are lowest. (2) DVI is strongly associated with CUBI (ρ = 0.842; *p* < 0.001), but this is ecological. (3) Within-region inequality accounts for 61.4% of total inequality. (4) Female education, sanitation, and women's empowerment show significant negative associations with CUBI, stronger at higher quantiles. (5) SDM indicates spatial dependence (ρ = 0.413), but not causal spillovers.

Overall, the evidence supports geographically differentiated approaches to child nutrition policy. However, the revised interpretation deliberately limits the empirical claims to what can be supported by cross-sectional, state-level NFHS-6 fact-sheet data. CUBI is a child anthropometric index, DVI is a separate exploratory dietary-vulnerability index, and the spatial models identify spatial association rather than causal diffusion.

## 4 Discussion

The present study provides a comprehensive assessment of nutritional inequality across Indian States and Union Territories using NFHS-6 (2023–24) data. By integrating composite indices, spatial autocorrelation, Theil decomposition, quantile regression, and spatial econometric models, the analysis moves beyond national averages to identify the multidimensional and geographically clustered nature of child undernutrition. The findings reveal substantial interstate heterogeneity, significant spatial clustering, dominant within-region inequality, and important ecological associations with dietary vulnerability, female education, sanitation, and women's empowerment. These findings have important implications for geographically targeted and multisectoral nutrition strategies, while requiring caution in interpreting ecological associations as individual-level causal relationships.

### 4.1 Persistent regional disparities and spatial clustering

The findings confirm marked interstate variation in stunting, wasting, underweight, and composite nutritional burden. National prevalence rates of 29.3% for stunting, 19.0% for wasting, and 31.8% for underweight mask substantial regional differences. Jharkhand, Bihar, and Gujarat emerge as the most nutritionally vulnerable states, whereas Goa, Kerala, and Tamil Nadu exhibit comparatively lower burdens. Significant positive spatial autocorrelation (Moran's *I* = 0.47, *p* < 0.01) indicates geographical clustering of nutritional deprivation, with hotspots concentrated in eastern and central India, forming an “east-central nutrition corridor.” This pattern is consistent with evidence from earlier NFHS rounds and suggests that structural conditions, including infrastructure, female literacy, sanitation, and dietary diversity, may also be spatially concentrated (24, 27).

The persistence of these clusters despite national progress is important. Previous evidence has similarly identified regional concentrations of undernutrition, indicating that structural factors remain relevant to geographical inequalities (17, 31). International comparisons show that India's spatial clustering is comparable to patterns reported in sub-Saharan Africa and higher than that observed in some Latin American settings (27, 28). Similar concentrations in other rapidly developing economies suggest that spatial nutritional inequality may accompany uneven economic and demographic transitions (3, 8).

### 4.2 Within-region inequality dominates nutritional disparities

The Theil decomposition shows that 61.4% of overall nutritional inequality originates from within-region variation, compared with 38.6% between broad geographic regions. These challenges strategies based solely on broad regional classifications and demonstrates substantial heterogeneity within regions. The western region contributes the largest share of total inequality (25.7%), while eastern India also shows considerable internal variation, particularly associated with persistent challenges in Bihar and neighboring states. In contrast, southern India records comparatively lower within-region variance and contribution to national inequality, potentially reflecting greater convergence in health systems and social development (14, 26).

The dominance of within-region inequality indicates that state-specific and district-level interventions may be more appropriate than uniform regional strategies. National programmes can establish common objectives, but resource allocation should reflect local nutritional conditions and structural constraints. Targeting disadvantaged districts within states may therefore generate greater nutritional improvements than uniform regional allocations (15, 23, 43).

### 4.3 Dietary vulnerability as a key correlate

The strong relationship between the Dietary Vulnerability Index (DVI) and Child Undernutrition Burden Index (CUBI) highlights the importance of dietary conditions. The Spearman's rank correlation is ρ = 0.842 (*p* < 0.001), indicating a strong positive ecological association. Bihar, Uttar Pradesh, and Assam, with high DVI scores, consistently experience greater nutritional vulnerability, whereas Kerala and Tamil Nadu record lower DVI values. Gujarat demonstrates that relatively stronger economic performance does not necessarily translate into favorable dietary conditions, ranking at the 89.2nd percentile in dietary vulnerability (32, 41).

These findings reinforce that nutrition depends not only on food quantity but also on dietary quality, diversity, and micronutrient adequacy (40, 42). Calorie-focused interventions may therefore be insufficient; agricultural diversification, food fortification, dietary diversification, and behavior-change communication are important complements to health interventions (22, 40). The DVI may also serve as a practical screening tool for identifying areas of nutritional vulnerability (48). However, the DVI–CUBI relationship is ecological and cannot establish that improving individual dietary diversity causes lower child undernutrition.

### 4.4 Structural correlates: education, sanitation, and women's empowerment

The ecological regression model explains 64% of state-level CUBI variation, with female education showing the strongest negative association (β = −0.412, *p* < 0.01). States with higher proportions of women completing at least 10 years of schooling consistently show lower CUBI scores, consistent with evidence on maternal education and child nutrition (22, 24, 39). At the macro level, female education may operate alongside better health infrastructure, household resources, economic participation, and social norms. These pathways, however, cannot be separated using aggregate data.

Sanitation also demonstrates a strong negative ecological association (β = −0.365, *p* < 0.01), supporting evidence linking sanitation and environmental conditions with child nutrition (11, 12). Women's status is similarly associated with lower CUBI (β = −0.281, *p* = 0.02), highlighting the importance of gender equity, financial inclusion, and women's autonomy (49). Urbanization shows a weaker association (β = −0.158, *p* = 0.07), potentially reflecting unequal access to healthcare, sanitation, and diversified food markets among peri-urban and informal populations (9, 13).

Collectively, these findings support a multisectoral understanding of nutrition encompassing education, sanitation, women's empowerment, urban planning, and health systems (3, 19, 21). However, these remain macro-level structural associations and should not be interpreted as individual-level behavioral mechanisms.

### 4.5 Quantile regression: heterogeneous associations across the burden distribution

Quantile regression demonstrates that ecological associations vary across the CUBI distribution. Female education has a stronger negative association in high-burden states (β = −0.487 at the 90th percentile) than in low-burden states (β = −0.346 at the 10th percentile), suggesting that education may be particularly relevant in disadvantaged contexts (22, 39). Sanitation shows relatively consistent associations across the distribution (β = −0.312 to −0.374), while women's status demonstrates a stronger association in high-burden states (β = −0.312) than in low-burden states (β = −0.218). The increase in pseudo-*R*^2^ from 0.58 to 0.67 indicates greater explanatory power among high-burden states.

These findings support place-based interventions that intensify investments in female education, sanitation, and women's empowerment in high-burden settings. Uniform strategies may be less efficient than approaches that account for differences across the burden distribution (14, 23, 29, 30). Nevertheless, these associations remain ecological and should not be interpreted as causal effects.

### 4.6 Rank stability and multidimensional deprivation

The rank stability matrix shows relatively consistent performance across nutritional indicators for Kerala, Tamil Nadu, Jharkhand, and Gujarat, suggesting that nutritional disadvantage is structural and multidimensional rather than indicator-specific (14, 15). Consequently, isolated interventions targeting only wasting, stunting, or underweight may be insufficient; comprehensive strategies addressing chronic and acute undernutrition simultaneously are required (19, 20).

Uttar Pradesh's high rank variance (17.6) indicates uneven performance across indicators and may reflect intra-state heterogeneity or differential programme implementation. This reinforces the need for district-level analysis to identify locations where specific forms of undernutrition are concentrated (31, 50).

### 4.7 Spatial spillover effects: evidence for spatial associations

The Spatial Durbin Model (SDM) indicates significant spatial associations in nutritional outcomes. The spatial autoregressive coefficient (ρ = 0.413, *p* < 0.01) shows that a state's CUBI is positively associated with that of neighboring states, reinforcing the observed spatial clustering (46). Female education demonstrates significant direct (β = −0.362, *p* < 0.01) and indirect (β = −0.184, *p* < 0.05) associations.

However, these spillover findings should be interpreted as exploratory spatial associations rather than causal diffusion. The ecological cross-sectional design and small sample (*N* = 36) do not permit conclusions about mechanisms such as knowledge diffusion, migration, or shared food systems. The observed associations may instead reflect shared unobserved characteristics, including regional economic conditions, cultural norms, infrastructure, or historical investments (24, 46). Indirect effects are also sensitive to spatial-weight specifications, requiring confirmation using larger samples, such as district-level data with *N* > 600, and longitudinal designs.

Sanitation and women's status show negative but non-significant indirect effects, potentially reflecting limited statistical power or more localized effects (11, 12). Although total effects exceed direct effects, these results should not be interpreted as definitive evidence of cross-state diffusion. Model comparison supports the SDM for capturing spatial dependence in nutritional outcomes (26, 31, 46).

### 4.8 Comparison with previous NFHS rounds

Although temporal change is not the primary focus, NFHS-6 estimates indicate modest improvement over NFHS-5, particularly in stunting, while changes in wasting and underweight remain comparatively limited (6, 25). However, substantial interstate disparities persist, with Kerala and Tamil Nadu continuing to perform favorably while several high-burden states remain disadvantaged. This persistence highlights the influence of initial conditions, cumulative investments, institutional capacity, and structural determinants on nutritional outcomes (7, 16, 17, 23).

The continued spatial clustering across survey rounds is concerning. Despite economic growth and multiple nutrition programmes, eastern and central India remain concentrated areas of nutritional disadvantage, suggesting uneven progress and persistent structural constraints. More intensive place-based strategies integrating education, sanitation, women's empowerment, dietary improvement, and health-system strengthening are therefore warranted (50, 51).

Overall, nutritional inequality reflects interconnected spatial, structural, dietary, and socioeconomic conditions. Within-region inequality supports district-sensitive strategies, while the DVI–CUBI association highlights dietary quality as an important ecological correlate. Female education, sanitation, and women's empowerment also show strong associations with nutritional outcomes. Although spatial modeling indicates correlated outcomes among neighboring states, the ecological design and limited sample require cautious interpretation. Geographically differentiated, multisectoral interventions are therefore needed, alongside district-level, individual-level, longitudinal, and quasi-experimental research to validate these relationships and clarify causal mechanisms.

### 4.9 Limitations and cautions on inference

Several limitations must be acknowledged:

**Ecological design and causal inference:** the ecological and cross-sectional design precludes causal inference; associations do not imply causation (14, 23). Findings should therefore be interpreted as macro-level structural associations rather than individual-level causal relationships. The ecological fallacy must be avoided, as state-level relationships may not apply to individuals. For example, higher female education at the state level does not necessarily imply better nutritional outcomes among individual children; individual-level and longitudinal analyses are required to establish causal mechanisms (24, 39).

**Data limitations:** the analysis uses state-level NFHS-6 fact-sheet data, limiting assessment of household- and individual-level determinants and obscuring intra-state heterogeneity. District-level fact sheets were unavailable at the time of analysis; future research should examine district-level disparities once released (31, 50).

**Anemia data exclusion:** the exclusion of Manipur due to data unavailability reduces geographic coverage. In addition, inconsistent availability of anemia indicators prevented their inclusion in CUBI. Thus, CUBI captures child anthropometric failure through stunting, wasting, and underweight and should not be interpreted as a comprehensive measure of nutritional deprivation (44, 45). Iron deficiency affects work capacity and human capital (52).

**Weighting and clinical severity:** equal weighting of CUBI components, although supported by sensitivity analysis, may not reflect differences in clinical severity (14, 20). CUBI should therefore be interpreted as a descriptive ranking tool rather than a clinical severity score.

**Reporting biases and small groups:** potential reporting biases cannot be ruled out, although standardized NFHS protocols mitigate this concern. Theil decomposition involving small regional groups also requires caution, although alternative groupings support the main finding that within-region inequality exceeds between-region inequality.

**Spatial model limitations:** the Spatial Durbin Model uses only *N* = 36 observations, limiting statistical power and potentially producing unstable estimates. Spillover effects are sensitive to spatial-weight specifications (46). These findings should therefore be treated as exploratory spatial associations, requiring confirmation through larger district-level datasets (*N* > 600) and longitudinal research.

## 5 Policy implications

The findings provide important directions for strengthening India's nutrition agenda under POSHAN Abhiyaan and Mission Poshan 2.0. The identification of Jharkhand, Bihar, and Gujarat as high-burden states highlights the need for geographically targeted resource allocation using the Child Undernutrition Burden Index (CUBI). State- and district-level CUBI mapping can support prioritization of maternal and child nutrition programmes, fortified supplementary nutrition, maternal health services, and intensified monitoring in vulnerable areas.

Female education shows the strongest ecological association with lower undernutrition, underscoring the need to integrate nutrition policy with education and women's empowerment. Expanding girls' secondary education, particularly in disadvantaged states, alongside self-help groups, financial inclusion, and nutrition-sensitive livelihoods, may contribute to improved child nutrition. Stronger associations in high-burden states further support prioritizing such investments, although individual-level research is required to establish causality.

The association between improved sanitation and lower nutritional burden reinforces convergence with the Swachh Bharat Mission. Policy should focus not only on infrastructure but also on sustained usage, hygiene practices, and behavioral change. Similarly, dietary vulnerability highlights the importance of food-system diversification. The Public Distribution System, ICDS, and school feeding programmes should strengthen access to pulses, millets, fortified foods, and other nutrient-rich, locally appropriate, and climate-resilient diets.

Since 61.4% of inequality occurs within regions, stronger coordination across health, education, food, sanitation, and social protection sectors is essential. State- and district-level nutrition convergence mechanisms and Poshan Tracker-based monitoring can improve accountability and identify emerging hotspots using CUBI and DVI indicators.

Finally, spatial spillover associations suggest potential benefits from regional cooperation, but these findings remain exploratory given the cross-sectional ecological design and small sample (*N* = 36). Future district-level and longitudinal evidence should validate these patterns. Cluster-based interventions across contiguous high-burden districts could subsequently strengthen coordinated action and accelerate progress toward national and global nutrition targets.

## 6 Conclusion

This study provides a multidimensional spatial assessment of nutritional inequality across 36 Indian States and Union Territories using NFHS-6 (2023–24) data. Findings reveal substantial regional disparities, with Jharkhand, Bihar, and Gujarat experiencing higher nutritional burdens, while Goa, Kerala, and Tamil Nadu show comparatively favorable outcomes. Significant spatial clustering (Moran's *I* = 0.47) highlights undernutrition hotspots in eastern and central India. Theil decomposition indicates that 61.4% of inequality occurs within regions, emphasizing state- and district-specific interventions. Quantile regression shows stronger associations of female education, sanitation, and women's empowerment in high-burden states. The Spatial Durbin Model (ρ = 0.413) indicates positive spatial spillovers, suggesting potential benefits from regional coordination. Dietary diversity remains an important pathway linking development and nutrition. As these are macro-level ecological associations, causal interpretation requires caution. Overall, geographically targeted, multisectoral policies are essential to reduce India's persistent nutritional inequalities.

### 6.1 Key findings

a Significant positive spatial autocorrelation (Moran's *I* = 0.47, *p* < 0.01) confirms clustering of undernutrition in eastern and central India, forming an “east-central nutrition corridor.”
b Within-region inequality (61.4%) dominates over between-region inequality (38.6%), supporting district-level targeting over broad regional strategies.
c Female education (β = −0.412), sanitation (β = −0.365), and women's empowerment (β = −0.281) show the strongest protective ecological associations, accounting for 64% of state-level CUBI variation.
d Quantile regression reveals stronger ecological associations of female education (β = −0.487) and women's empowerment (β = −0.311) in high-burden states, justifying targeted investments.
e Spatial spillover associations (ρ = 0.413) demonstrate cross-state influences, suggesting the potential for regional coordination of nutrition policies, though this requires confirmation with more robust evidence.

**Extensions and wider implications:** the findings have broader relevance for countries experiencing substantial regional nutritional disparities. The combined use of composite indices, inequality decomposition, and spatial econometrics provides a transferable framework for identifying high-priority areas. Persistent spatial clustering despite national progress highlights the limitations of aggregate monitoring and reinforces the importance of subnational approaches for achieving the SDGs. The strong DVI–CUBI association further emphasizes food-system transformation through dietary diversification, agricultural diversification, and food fortification alongside health interventions.

**Future research directions:** future studies should use forthcoming NFHS-6 district-level data to examine intra-state disparities and temporal changes. Larger samples (*N* > 600), longitudinal and quasi-experimental designs are needed to validate spatial spillovers and distinguish diffusion from shared confounding factors. Individual-level analyses should examine maternal education, sanitation, and women's empowerment, while incorporating anemia, climate–nutrition linkages, and mixed-methods evidence. Overall, reducing nutritional inequality requires evidence-based, multisectoral, geographically targeted interventions prioritizing vulnerable populations.

## Data Availability

The datasets presented in this study can be found in online repositories. The names of the repository/repositories and accession number(s) can be found below: the data used in this study are publicly available from the International Institute for Population Sciences (IIPS) NFHS-6 fact sheets (https://www.nfhsiips.in/nfhsuser/index.php). The processed analytical datasets, R and Python code used for spatial analysis, Theil decomposition, and composite index construction are available from the corresponding author upon reasonable request.
