# Supplementary material for: State-level nutritional disparities and policy priorities in India
Source: Front Nutr. 2026 Sep 8;13:1902541. doi: 10.3389/fnut.2026.1902541 (PMC13595373; doi:10.3389/fnut.2026.1902541)
Supplement: Supplementary file 1 [file Data_Sheet_1.pdf]

APPENDIX

Supplementary Table S1. Complete Regional Classification of All 35 States and Union Territories Used in Theil Decomposition

Region	States/ Union Territories	Number of Units
North	Chandigarh, Delhi, Haryana, Himachal Pradesh, Jammu & Kashmir, Ladakh, Punjab, Uttarakhand	8
South	Andhra Pradesh, Karnataka, Kerala, Tamil Nadu, Telangana	5
East	Bihar, Jharkhand, Odisha, West Bengal	4
West	Goa, Gujarat, Rajasthan	3
Central	Chhattisgarh, Madhya Pradesh, Maharashtra, Uttar Pradesh, Dadra & Nagar Haveli and Daman & Diu	5
Northeast	Arunachal Pradesh, Assam, Manipur, Meghalaya, Mizoram, Nagaland, Sikkim, Tripura	8
Union Territories (Consolidated)	Andaman & Nicobar Islands, Lakshadweep, Puducherry	3
Total	States and Union Territories	36

Supplementary Table S2. Region-Specific Theil Decomposition Results Under the Revised Regional Classification

Region	Number of Units	Mean CNBI	Within-Region Theil Index	Share of National Inequality (%)
North	8	-0.42	0.026	10.8
South	5	-0.86	0.021	8.1
East	4	0.72	0.038	17.6
West	3	0.39	0.031	11.5
Central	5	0.58	0.043	14.2
Northeast	8	-0.36	0.029	9.2
Union Territories	3	-0.91	0.018	4.8
Within-Region Component	36	–	0.206	61.4
Between-Region Component	36	–	0.129	38.6
Overall Theil Index	36	–	0.335	100.0

Supplementary Table S3. Principal Component Analysis Results: Eigenvalues, Component Loadings, and Percentage of Variance Explained

Panel A. Eigenvalues and Explained Variance				
Principal Component	Eigenvalue	Difference	Proportion of Variance (%)	Cumulative Variance (%)
PC1	2.352	1.910	78.40	78.40
PC2	0.442	0.236	14.73	93.13
PC3	0.206	–	6.87	100.00

Panel B. Factor Loadings				
Variable	PC1	PC2	PC3	Communality
Child Stunting (%)	0.914	-0.118	0.388	0.849
Child Wasting (%)	0.781	0.462	-0.419	0.823
Child Underweight (%)	0.936	-0.071	-0.344	0.881

Panel C. Sampling Adequacy and PCA Diagnostics

Diagnostic Test	Statistic	Interpretation
Kaiser–Meyer–Olkin (KMO)	0.781	Good sampling adequacy
Bartlett's Test of Sphericity (χ^2)	48.67	Significant
Degrees of Freedom	3	—
p-value	<0.001	Correlation matrix suitable for PCA
Cronbach's Alpha	0.842	High internal consistency
Composite Reliability	0.879	Excellent reliability
